# Structural and Biochemical Characterization of Botulinum Neurotoxin Subtype B2 Binding to Its Receptors

**DOI:** 10.3390/toxins12090603

**Published:** 2020-09-17

**Authors:** Jonathan R. Davies, Geoffrey Masuyer, Pål Stenmark

**Affiliations:** 1 Department of Biochemistry and Biophysics, Stockholm University, SE-106 91 Stockholm, Sweden; jonathan.davies@dbb.su.se; 2 Department of Pharmacy and Pharmacology, Centre for Therapeutic Innovation, University of Bath, Bath BA2 7AY, UK; 3 Department of Experimental Medical Science, Lund University, SE-221 00 Lund, Sweden

**Keywords:** *Clostridium botulinum*, botulism, botulinum neurotoxin, BoNT/B, synaptotagmin, ganglioside

## Abstract

Botulinum neurotoxins (BoNTs) can be used therapeutically to treat a wide range of neuromuscular and neurological conditions. A collection of natural BoNT variants exists which can be classified into serologically distinct serotypes (BoNT/B), and further divided into subtypes (BoNT/B1, B2, …). BoNT subtypes share a high degree of sequence identity within the same serotype yet can display large variation in toxicity. One such example is BoNT/B2, which was isolated from *Clostridium botulinum* strain 111 in a clinical case of botulism, and presents a 10-fold lower toxicity than BoNT/B1. In an effort to understand the molecular mechanisms behind this difference in potency, we here present the crystal structures of BoNT/B2 in complex with the ganglioside receptor GD1a, and with the human synaptotagmin I protein receptor. We show, using receptor-binding assays, that BoNT/B2 has a slightly higher affinity for GD1a than BoNT/B1, and confirm its considerably weaker affinity for its protein receptors. Although the overall receptor-binding mechanism is conserved for both receptors, structural analysis suggests the lower affinity of BoNT/B2 is the result of key substitutions, where hydrophobic interactions important for synaptotagmin-binding are replaced by polar residues. This study provides a template to drive the development of future BoNT therapeutic molecules centered on assessing the natural subtype variations in receptor-binding that appears to be one of the principal stages driving toxicity.

## 1. Introduction

Botulinum neurotoxins (BoNTs) are bacterial toxins produced predominantly by *Clostridium botulinum*. In their active form, BoNTs are composed of a di-chain polypeptide consisting of a ~100 kDa heavy chain and ~50 kDa light chain (LC) which remain linked by a single disulfide bond [1]. BoNTs present three modular domains that each play a role in mediating the steps necessary for toxicity. The heavy chain contains two domains: the receptor-binding domain (H_C_) which is responsible for targeting neuronal cells at the presynaptic neuromuscular junction, and the translocation domain (H_N_) which transfers the LC across the endosomal membrane within the cytosol. The LC consists of a single Zn-dependent metalloprotease domain which cleaves a neuronal SNARE (soluble NSF (N-ethylmaleimide-sensitive factor) attachment protein receptors) protein, and results in inhibition of acetylcholine release from nerve cells, thereby leading to flaccid paralysis.

Many distinct BoNTs have been identified which are categorized into multiple BoNT serotypes (BoNT/A to /G, /X) based on the cross-reactivity of polyclonal neutralizing antibodies [2,3]. The neuronal receptors for most serotypes have been identified. BoNT/A, /D, /E, and /F utilize the synaptic vesicle protein 2 (SV2) family as their protein receptor. BoNT/B, /G, and /DC instead bind to either Synaptotagmin I or II (SytI or SytII) [4,5,6,7,8,9,10,11]. In addition, all serotypes bind gangliosides which are found in abundance on neuronal membranes and offer a first cell-surface anchor that promotes binding to the protein receptor [12]. Recently, an updated model was suggested in which a preassembled ganglioside-synaptotagmin complex constitutes the high-affinity BoNT/B receptor [13].

Within each serotype, BoNTs can be further subcategorized into subtypes based on their amino acid sequences. Each novel subtype is generally agreed to differ by more than 2.6% [14]. Despite their high sequence identities, previous characterization of similar BoNT subtypes has revealed distinct intoxication properties which may have implications for future therapeutic applications [15]. In particular, in vivo data collected for BoNT/A subtypes 1 to 5 (BoNT/A1 to /A5) highlighted significant intoxication differences, such as onset time and duration of action [16]. The majority of commercially available BoNT products are based on BoNT/A1. The single exception is the product, rimabotulinumtoxinB (Myobloc^®^), which is based on BoNT/B1. However, BoNT/B1 suffers from a low affinity to the human form of Synaptotagmin II (hSytII) when compared with the mouse form due to a single residue difference between the species [11,17,18]. The low affinity of BoNT/B1 in humans requires a larger dose to achieve the same effect as a BoNT/A1-based product. There are currently over 40 known BoNT subtypes of the various serotypes, suggesting there is potential for therapeutic improvement following further exploration of this repertoire.

**Figure 1:**
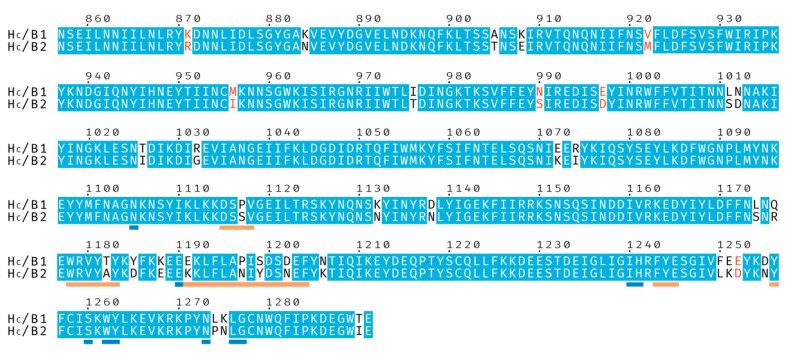
Sequence alignment of the binding domains from botulinum neurotoxins BoNT/B1 and BoNT/B2. Identical residues are shown with a blue background, and similar residues with red characters. Residues underlined in orange are involved in, or neighboring, the Synaptotagmin-binding site and residues underlined in blue are involved in, or neighboring, the ganglioside-binding site.

BoNT/B2 from *Clostridium botulinum* strain 111 was first identified in a case of infant botulism [19] and initial characterization found its toxicity to be approximately 10 times lower than that of BoNT/B1 [20]. Subsequent studies have confirmed the reduced toxicity and suggested that it is likely due to receptor-binding differences [21,22,23]. The H_C_ of BoNT/B2 shares 92% sequence identity with BoNT/B1 (Figure 1). We therefore decided to further investigate the molecular mechanisms behind these observations. Here, we present the crystal structures of BoNT/B2 H_C_ bound to its GD1a and synaptotagmin receptors and, using previously determined BoNT/B1 receptor complex structures [9,24,25], compare structural differences between the BoNT/B1 and /B2 H_C_ domains.

## 2. Results and Discussion

### 2.1. Crystallisation of H_C_/B2

**Figure 2:**
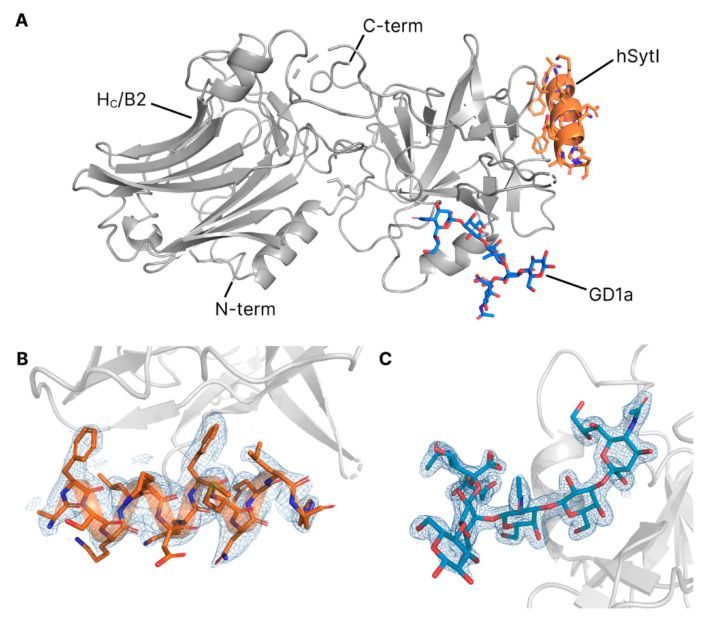
(**A**) Merge of the two crystal structures of H_C_/B2 (grey), determined here in complex with hSytI (orange) and GD1a (blue), respectively. (**B**) Electron density surrounding the hSytI peptide, contoured to 1.2 σ. (**C**) Electron density surrounding the GD1a ganglioside, contoured to 1.2 σ.

The purified H_C_/B2 protein after tag-cleavage with tobacco etch virus protease (TEV) consisted of residues 857 to 1291 of the full-length BoNT/B2 protein with an additional three N-terminal linker residues. Attempts were made to co-crystallize H_C_/B2 in combination with both GD1a and Synaptotagmin, like has been previously reported for H_C_/B1 [24], but we were unable to obtain crystals containing both receptors together. Instead, only crystals containing H_C_/B2 in complex with hSytI were produced in the space group P2_1_2_1_2_1_, which diffracted to a resolution of 2.5 Å (Table 1). Electron density can be seen for residues 46 to 59 of hSytI but not for residues either side, indicating some flexibility (Figure 2). Separate crystallization experiments containing only GD1a and H_C_/B2 were successful in producing crystals in the space group C121, containing the ganglioside-bound complex which diffracted to a resolution of 1.8 Å (Table 1). The electron density for the entire GD1a carbohydrate is well defined (Figure 2). The overall structure of H_C_/B2 is highly similar to H_C_/B1 and its coordinates differ by a root-mean-square deviation (rmsd) of 0.60 Å for the GD1a-bound structure (PDB ID 6ZVM) and 0.62 Å for the hSytI-bound structure (PDB ID 6ZVN).

### 2.2. Ganglioside-Binding Properties of H_C_/B2

#### 2.2.1. Molecular Details of GD1a Binding

**Figure 3:**
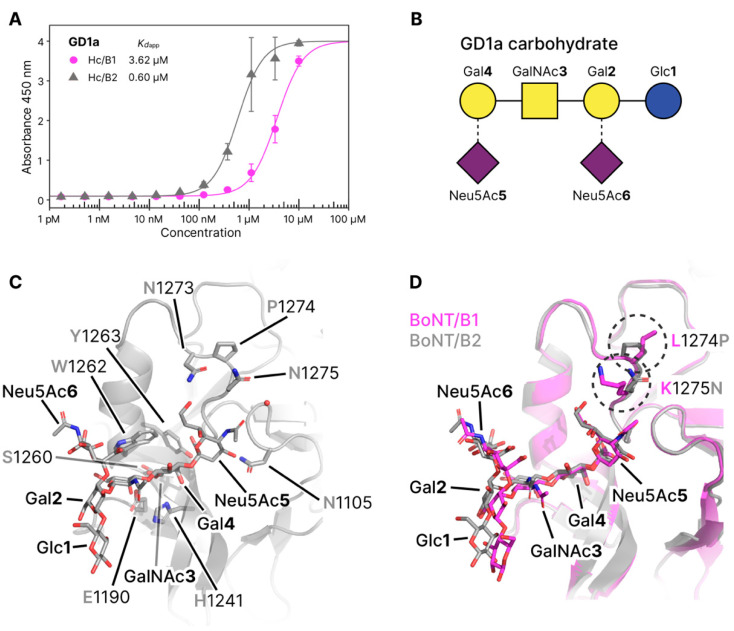
Ganglioside-binding. (**A**) GD1a binding of Hc/B1 and /B2. Apparent *Kd* values are 3.62 ± 0.11 * µM for H_C_/B1 and 0.60 ± 0.04 * for H_C_/B2. Assays were performed in triplicate, * standard error. (**B**) Glycoblock schematic representation (McNicholas 2016) of the GD1a carbohydrate used for crystallization experiments. (**C**) Close view of the GBS from BoNT/B2 with GD1a bound (this publication), water involved in binding as a red sphere. (**D**) Overlay of H_C_/B1 (magenta) in complex with GD1a (PDB 4KBB) and H_C_/B2 (grey) in complex with GD1a (this publication). Amino acid differences between the two proteins in this region are circled.

The single ganglioside binding site (GBS) of BoNT/B1 has previously been identified through a combination of mutagenesis, crystallization, and sequence homology [10,24,26]. Like BoNT/B1, /B2 contains a conserved SxWY motif in the putative GBS. It has also been shown that BoNT/B1 has a preference for complex gangliosides such as GT1b and GD1a [12]. The structure of the GBS is highly conserved with sequence differences only noted both sides of the GBS (Figure 1). Our GD1a-bound structure of the H_C_ domain from BoNT/B2 reveals a highly similar binding conformation of both GD1a and the H_C_ domain when compared to BoNT/B1 (Figure 3). Four of the five monosaccharides within the GD1a carbohydrate (N-acetylneuraminic acid [Neu5Ac**5**], galactose [Gal**4**], N-acetylglucosamine [GalNAc**3**], and Neu5Ac**6** directly interact with the GBS. Neu5Ac**5** forms hydrogen bonds to N1273, N1275, G1104, N1105, G1277, and Y1263. Gal**4** hydrogen bonds with His1241, I1240, S1260, and E1190, and also forms stacking interactions with W1262. GalNAc**3** forms a single hydrogen bond to E1190, and Neu5Ac**6** forms a single hydrogen bond to W1262. Neither Gal**2** or Glc**1** form any direct bonds with the protein. When comparing with the B1-GD1a structure, the central Gal4 superposes extremely well, but minute variations in the position of other monosaccharides are a potential result of differences in surrounding loops.

One such difference is residue 1275 which is a Lys in B1 and Asn in B2. In our ganglioside-bound crystal structure, we observed a potential water-mediated interaction between N1275 and Neu5Ac**5** which may result in improved binding affinity. The orientation of the acetyl group of Neu5Ac**5** could not be determined with confidence from the crystallographic data alone. The structure was refined separately with the acetyl in both orientations, however the temperature factor (B-factor) for the oxygen atom was always higher than for the methyl, suggesting the presence of both orientations. Adjacent to N1275 is P1274 which replaces a Leu in B1 and likely provides more rigidity to the loop in an orientation that could favor interactions with the Neu5Ac**5** moiety. Of note, one of the sequence variations observed between B1 and B2 is in a charged loop (residues 1187-1191) that connects the GBS to the Syt-binding site (KKEEEK in B1 and KEEEKK in B2). However, despite the proximity of position 1188 to the GBS, it does not affect interactions of GalNAc3, which shows a conserved hydrogen bonding pattern between B1 and B2.

#### 2.2.2. GD1a Binding Assay

To determine whether the reduced toxicity reported for BoNT/B2 is a result of differences in ganglioside binding, an assay similar to previously described protocols was used [27,28]. Here the polysialoganglioside GD1a was immobilized to the plate surface, and binding of the H_C_ domains from BoNT/B1 and /B2 were directly compared. GD1a was chosen to allow direct comparison with the crystal structure and B1. We observed a six-fold increase in binding toward GD1a by B2 (*Kd*_app_ B2 = 0.60 µM) compared to B1 (*Kd*_app_ B2 = 3.62 µM) (Figure 3). Observations from the crystal structures suggest an overall conserved binding mechanism, however the minor differences described above may explain this variation in affinity.

Previously, single point mutations of the GBS [22] suggested that while the conserved SxWY motif is necessary for toxicity, alterations of surrounding residues (1186, 1189, 1191, and 1260) did not significantly impact the overall binding affinity to the SytII/ganglioside complex. It is therefore unlikely that ganglioside binding is contributing to the lower toxicity of B2 compared to B1.

### 2.3. Synaptotagmin-Binding Properties of H_C_/B2

#### 2.3.1. Molecular Details of Syt-Binding

The binding mechanism between BoNT/B1 and synaptotagmin is well-characterized [8,9] and revealed that Syt forms a short helix that binds to a hydrophobic groove within the binding domain of BoNT/B, and adjacent to the GBS [24]. Here we show that hSyt1 binds to B2 by forming a helix at the same location, with its backbone superposing well with the previously determined structure of H_C_/B1 in complex with hSyt1 (PDB 6G5K [25]), but with significant local variations.

**Figure 4:**
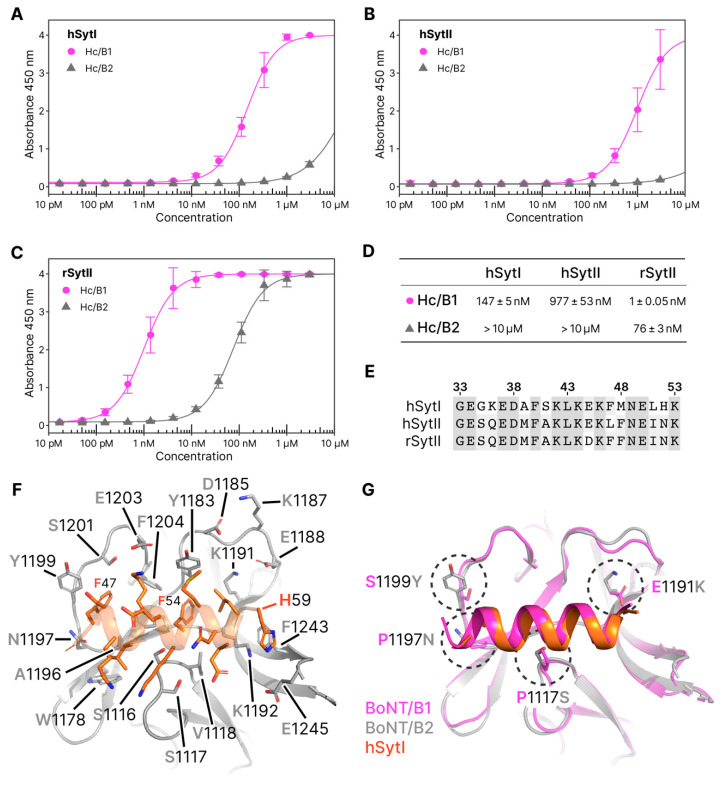
Synaptotagmin-binding. hSytI (**A**), hSytII (**B**), and rSytII (**C**) binding curves against H_C_/B1 and H_C_/B2. Assays were performed in triplicate. (**D**) Apparent K*d* (K*d_app_*) values for each of the fitted binding assays, with standard error. (**E**) Partial alignment of hSytI, hSytII, rSytII (sequence numbering of hSytI). (**F**) Overview of the Syt binding site of BoNT/B2, with hSytI bound. Residues involved or surrounding the site are labelled. (**G**) Superposition of the binding site from H_C_/B1(PDB 4KBB) and H_C_/B2. The position of hSytI is displayed in orange. Residue differences between the two H_C_ proteins are circled and labelled.

There are four residue differences between BoNT/B1 and BoNT/B2 which are in close proximity to the bound Syt peptide: P1117S, P1197N, E1191K, S1199Y (Figure 4). The change from two hydrophobic proline residues to two polar residues (Ser and Asn) is likely to result in a significant change in binding affinity as both of these residues are found neighboring the conserved and central Phe residues of Syt, known to be key elements of the hydrophobic binding mechanism [8,9]. Substitution of the two prolines (1117 and 1197) in B1 may also provide more flexibility to the B2 loops that would favor a faster dissociation rate. In BoNT/B2, this loss of hydrophobic interaction is replaced by electrostatic contacts between S1117 and N1197 and the peptide backbone of hSytI. Conversely, the change from S1199 in BoNT/B1 to Y1199 in BoNT/B2 would be expected to provide increased affinity for the peptide via aromatic interactions with F47. Here we observed that both N1197, via its hydrogen bond with F47, and Y1199 contribute to shifting the N-terminal part of the hSytI backbone slightly further away (1Å) compared to its position when bound to B1.

Remarkably, positions 1191 and 1199 were recently identified as sites that could be mutated to engineer a BoNT/B^MY^ toxin with improved affinity for its human synaptotagmin receptors, which also resulted in enhanced efficacy in preclinical models [18,25]. Mutations E1191M and S1199Y were shown to have an optimum effect when combined, allowing the C-terminal hydrophobic side chains of hSytI (L58) to interact deeper within the hydrophobic pocket of BoNT/B^MY^. In BoNT/B2 however, substitution of a negative (Glu) to a positive (Lys) side chain at position 1191 likely decreases the affinity of B2 for its receptor. Interestingly, in B2, K1191 makes a salt bridge with E1988 of the charged loop mentioned above (residues 1187-1191), that separates the two receptor-binding sites. This is reciprocal in B1 where E1191 makes a salt bridge with K1988, suggesting that the ‘conserved’ bond may be important for stability of this loop. In addition, although the side chain of Y1185 in B1 does not appear to take part in the Syt interaction, substitution for the polar D1185 in B2 may slightly hinder binding on this side of the binding pocket as well.

#### 2.3.2. Synaptotagmin-Binding Assay

To determine whether sequence differences within the synaptotagmin-binding site affect affinity, we performed receptor-binding assays similar to the ganglioside assay described previously. Three different GST-tagged Synaptotagmin peptides were immobilized to the assay plates in order to compare binding of H_C_/B1 and H_C_/B2: human SytI residues 1 to 53 (hSytI), human Syt II residues 1 to 57, (hSytII), and rat Syt II residues 1 to 60 (rSytII).

We observed a significantly higher affinity (over two orders of magnitude) for H_C_/B1 to each of the Syt proteins than for H_C_/B2 (Figure 4). For our hSytI and hSytII assay, the affinity of H_C_/B2 was too low to confidently determine the K*d_app_*. At the protein concentrations required to fit a full binding response, effects such as protein aggregation become detrimental to the assay.

There is a significant difference between the affinity values presented here and the ones established previously with other techniques such as isothermal titration calorimetry (ITC) or bio-layer interferometry (BLI) [8,18,23,29], and this is inherent to assay conditions and the choice of constructs used. Nevertheless, our data are consistent with the previous assertion that H_C_/B1 has a higher selectivity for rSytII (K*d_app_* = 1 nM) over hSytI (K*d_app_* = 147 nM), and hSytII (K*d_app_* = 977 nM) [11,17,18,29]. A trend that is also observed with H_C_/B2 (rSytII > hSytI > hSytII).

Although our receptor-binding data present a larger variation between B2 and B1, they support the comparison of BoNT/B subtypes by Kohda et al. [23], which showed that B2 has a significantly lower toxicity compared to B1 and B6, and associated this result with the lower Syt-binding capability of the B2 subtype. This is in agreement with the recent observations on engineered BoNT molecules consisting of the chimeric A/B serotypes [30,31], or of BoNT/B mutants with improved affinity for synaptotagmin [25]; that the protein receptor-binding function is key to the modulation of potency. Other functional steps of the toxin activity, such as the rate of translocation may also modulate toxicity and explain some of the variation between B1 and B2 (the translocation domain shares 95.4% sequence identity). The catalytic domain however is highly conserved (99.8% identity) and less likely to be involved in the difference of potency between B1 and B2, as also suggested by a recent report showing that an engineered B1 mutant, with higher catalytic efficiency, did not result in increased potency in physiological systems [32].

Interestingly, in vitro and ex vivo assays performed with the progenitor M complex of BoNT/B2, isolated from *C. botulinum* strain BL6 [33], which includes BoNT/B2 in complex with the non-toxic-non-hemagglutinin (NTNH) component, showed a remarkably higher potency compared to the equivalent B1 complex or the pure B1 toxin. Further characterization of the role of NTNH in the toxicity of the BoNT/B2 strains and functional comparison with the toxin on its own will be an interesting avenue for future research and may provide further therapeutic tools beyond the recombinant pure toxins.

Remarkably, the crystal structures presented here indicate that BoNT/B2 has conserved a similar overall receptor-binding mechanism to BoNT/B1, despite the significant variation in toxicity between the two subtypes. Whilst the ganglioside-binding site is near identical, the main differences appear at the synaptotagmin-binding site. Although the backbone of the hSytI peptide occupies a position similar to the one when bound to B1, we identified several amino acid substitutions that cause a loss of hydrophobic interactions between B2 and Syt, thus affecting the overall affinity for the receptor. Two of these sites, 1191 and 1199, were previously identified as locations in B1 that could be mutated to provide enhanced SytII-binding using a bacterial adenylate cyclase two-hybrid method, alongside a saturation mutagenesis screen [18]. Mutations to bulky hydrophobic amino acids (Met and Tyr, respectively) resulted in an over 20 times higher affinity for hSytII. In B2, the presence of Y1199 is not enough to compensate for the loss of affinity resulting from substitutions P1117S, P1197N, and E1191K.

Altogether our data provide the molecular basis behind the weaker affinity of BoNT/B2 for the synaptotagmin receptors, which likely contributes to the lower toxicity of this subtype. It highlights how the general mechanism of synaptotagmin receptor-binding can remain conserved yet offer considerable variations in affinity, caused by key hydrophobic substitution within the Syt-binding pocket. Our study suggests that a thorough structure-function analysis of the BoNT subtypes, in particular of their receptor-binding properties, may lead to the identification of natural toxins or engineered variants with enhanced therapeutic potential.

## 3. Materials and Methods

### 3.1. Constructs

DNA encoding H_C_/B1 (strain: Okra, BoNT/B1 residues 857 to 1291 (UniProtKB: B1INP5)) and H_C_/B2 (strain: 111, BoNT/B2 residues 857 to 1291 (UniProtKB: Q8GR96)) were synthesized by Genscript (Piscataway, NJ, USA) and cloned into an modified pET28a(+) vector, designed to express proteins containing an N-terminal 6xHis-tag, FLAG-tag, and TEV protease cleavage site. Three GST-tagged synaptotagmin constructs were designed and the DNA encoding each were synthesized by Genscript (Piscataway, NJ, USA) with flanking BamHI and XhoI restriction sites for cloning into the pGEX-5X-1 vector. For each construct, the N-terminal region (hSytI residues 1–53 [UniProtKB: P21579], hSytII residues 1–57 [UniProtKB: Q8N9I0], and rSytII residues 1–60 [UniProtKB: P29101]) were synthesized with an N-terminal 6xHis-tag after the BamHI restriction site for protein purification. All protein sequences are provided in Supplementary Materials.

### 3.2. Protein Expression and Purification

All proteins were expressed in TB media, inoculated using BL21 cells transformed with the respective vector. Cultures were grown in a LEX bioreactor (Epiphyte Three Inc. Toronto, ON, Canada) at 37 °C. When the OD600 reached 0.7, the temperature was reduced to 18 °C, and protein expression induced through the addition of 1 mM IPTG. Cells were grown for a further 18 h before harvesting by centrifugation.

For the H_C_/B1 and H_C_/B2 proteins, cells were resuspended in 50 mM HEPES pH 7.4, 200 mM NaCl, 20 mM Imidazole, and lysed using two passes of an Emulsiflex-C3 (AVESTIN Europe, GmbH, Mannheim, Germany) at 20 kPSI. The lysate was clarified by centrifugation at 50,000× *g* for 30 min before loading onto a pre-equilibrated 5 mL HisTrap HP column (GE Healthcare, Uppsala, Sweden). Purified protein was eluted using a linear gradient to 50 mM HEPES pH 7.4, 200 mM NaCl, and 500 mM Imidazole over 20 column volumes. Fractions containing the target H_C_ were pooled and further purified using a Superdex200 26/600 column (GE Healthcare, Uppsala, Sweden), pre-equilibrated using 50 mM HEPES pH 7.4, 200 mM NaCl. Prior to crystallization experiments with hSytI and GD1a, the N-terminal His-tag and FLAG-tag of H_C_/B2 were removed using TEV protease at 20 °C. Remaining His-tagged H_C_/B2 and TEV protease were removed by flowing the sample through a HisTrapHP, and untagged H_C_/B2 was eluted using 50 mM HEPES pH 7.4, 200 mM NaCl, 25 mM Imidazole.

For the Syt proteins, cells were resuspended in 100 mM HEPES, 500 mM NaCl, 10% glycerol, 10 mM imidazole, 0.5 mM TCEP, pH 8.0 and lysed by pulsed sonication (4s/4s 4 min, 80% amplitude). The sonicated lysate was clarified by centrifugation (20 min at 49,000× *g*) and the supernatant was filtered through 0.45 µm filters prior to purification. Clarified lysate was loaded onto a pre-equilibrated 5 mL HisTrap HP column (GE Healthcare, Uppsala, Sweden), and eluted using 20 mM HEPES, 500 mM NaCl, 10% glycerol, 500 mM imidazole, 0.5 mM TCEP, pH 7.5. Eluted protein was further purified using a Superdex200 16/600 column (GE Healthcare, Uppsala, Sweden), pre-equilibrated using 20 mM HEPES, 300 mM NaCl, 10% glycerol, 0.5 mM TCEP, pH 7.5.

### 3.3. Crystallisation

Prior to crystallization of H_C_/B2 in complex with hSytI, a solution containing untagged-H_C_/B2 (190 µM), hSytI (residues 33 to 53, synthesized by Genscript, Piscataway, NJ, USA. 1 mM), and GD1a (5 mM) was made. Crystals were obtained at 21 °C using the sitting-drop vapor diffusion method where 100 nL of the protein solution was mixed with 100 nL reservoir solution, consisting of 0.1 M imidazole pH 8.0, 10% (*w*/*v*) PEG 8000 from the JSCG+ screen.

To obtain crystals of H_C_/B2 in complex with GD1a, a solution containing H_C_/B2 (190 µM) and GD1a (1 mM) was prepared. Crystals were obtained at 21 °C using the sitting-drop vapor diffusion method, where 100 nL of the protein solution was mixed with 100 nL reservoir solution consisting of 0.16 M calcium acetate hydrate, 0.08 M MES pH 6.5, 14.4% (*w*/*v*) PEG 8000, and 20% (*v*/*v*) glycerol from the JSCG+ screen.

### 3.4. X-Ray Data Collection and Data Reduction

X-ray diffraction data were collected from single crystals at 100 K on beamline I04 at Diamond Light Source (UK) using an Eiger2 XE 16M detector (Dectris, Baden, Switzerland). Diffraction data were indexed and integrated using DIALS [34]. Data were scaled and merged using AIMLESS [35] from the CCP4 suite [36]. An initial model of H_C_/B2 was generated with Phyre2 [37] for phasing by molecular replacement using Phaser [38]. The working models were refined using REFMAC5 [39] and manually adjusted with COOT [40]. The conformation of the GD1a within the crystallographic model was validated using Privateer [41]. Protein validation was performed with MOLPROBITY [42]. Crystallographic data statistics are summarized in Table 1. The atomic coordinates and structure factors (PDB ID 6ZVM and 6ZVN) have been deposited in the Protein Data Bank (http://wwpdb.org). Protein structure figures were rendered with PyMOL (Schrödinger, LLC, NY, USA).

### 3.5. Synaptotagmin-Binding Assay

Purified GST-tagged synaptotagmin was diluted to 10 µg/mL in 0.1 M Tris-HCl pH 8.0 and 100 µL used to coat each well of a Nunc-Immuno 96-well plate (Merck M9410) overnight at 4 °C. Wells were washed with 200 µL phosphate-buffered saline (PBS) containing 0.05% (*v*/*v*) Tween-20 (PBS-T) and 0.1% (*w*/*v*) bovine serum albumin (BSA). Non-specific binding sites were blocked by incubation of each well for 1 h at 22 °C with 200 µL PBS containing 2% (*w*/*v*) BSA. After blocking, each well was washed with 200 µL PBS-T. Binding assays were performed in 100 µL PBS-T with 0.1% (*w*/*v*) BSA containing FLAG-tagged H_C_/B proteins in a concentration series. The binding assay was incubated for 1h at 21 °C before washing each well three times with PBS containing 0.1% (*w*/*v*) BSA. To detect bound H_C_/B protein, wells were incubated with monoclonal anti-FLAG HRP-conjugated antibody (Merck, A8592) diluted 1:20000 into 100 µL PBS containing 0.1% (*w*/*v*) BSA. Wells were washed again three times with 200 µL PBS containing 0.1% (*w*/*v*) BSA and bound antibody was detected using 100 µL Ultra TMB-ELISA substrate solution (ThermoFisher, 34029, Stockholm, Sweden). The reaction was stopped by the addition of 100 µL 0.2 M H_2_SO_4_, and the absorbance at 450 nm was determined using a plate reader. Results were analyzed with Prism (GraphPad, La Jolla, CA, USA), using a non-linear logistic binding fit.

### 3.6. Ganglioside-Binding Assay

Ganglioside GD1a was purchased from Carbosynth (Compton, UK). GD1a was dissolved in DMSO at a stock concentration of 2.5 mg/mL. The GD1a stock was diluted in methanol to reach a final concentration of 2.5μg/mL; 100 μL (0.25 μg) was applied to each well of a 96-well PVC assay plates. After evaporation of the solvent at 21 °C (overnight), the wells were washed (3 times) with 200 μL of PBS/0.1% (*w*/*v*) BSA. Nonspecific binding sites were blocked by incubation for 2 h at 21 °C in 200 μL of PBS/2% (*w*/*v*) BSA. Binding assays were performed in 100 μL of PBS/0.1% (*w*/*v*) BSA per well for 2 h at 4 °C containing the H_C_/B samples (serial 3-fold dilution ranging from 10 μM to 60 pM). Following incubation, wells were washed 3 times with PBS/0.1% (*w*/*v*) BSA and then incubated with monoclonal anti-FLAG HRP-conjugated antibody (Merck, A8592) diluted 1:20000 into 100 µL PBS containing 0.1% (*w*/*v*) BSA for 1 h at 4 °C. Finally, after three washing steps with PBS/0.1% (*w*/*v*) BSA, bound samples were detected using Ultra TMB substrate solution (ThermoFisher, 34029) (100 μL/well). The reaction was terminated after incubation for 5 min at 21 °C by addition of 100 μL of 1M sulphuric acid. Absorbance at 450 nm was measured with a Tecan Infinite 200 (Männedorf, Switzerland). Results were analyzed with Prism (GraphPad, La Jolla, CA, USA), using a non-linear logistic binding fit.

## Figures and Tables

**Table 1:** Crystallographic Data Collection and Refinement.

	H_C_/B2-GD1a	H_C_/B2-hSytI
Data Collection		
Beamline	DLS-I04	DLS-I04
Wavelength (Å)	0.9795	0.9795
Space group	C 121	P 2_1_2_1_2_1_
Cell dimensions:		
*a*, *b*, *c* (Å)	139.6, 56.2, 76.8	57.6, 82.5, 106.7
α, β, γ (°)	90.0, 122.0, 90.0	90.0, 90.0, 90.0
Resolution (Å)	63.7–1.8 (1.84–1.80) ^1^	65.2–2.5 (2.6–2.5) ^1^
No. total/unique reflections	298592/46758	201407/18223
R_meas_	0.109 (0.777) ^1^	0.267 (2.687) ^1^
R_pim_	0.042 (0.356) ^1^	0.082 (0.799) ^1^
CC_1/2_	0.996 (0.682) ^1^	0.949 (0.447) ^1^
<I/σ(I)>	10.6 (2.3) ^1^	5.7 (1.0) ^1^
Completeness (%)	99.6 (96.2) ^1^	99.7 (99.9) ^1^
Redundancy	6.4 (4.5) ^1^	11.1 (11.0) ^1^
**Refinement**		
R_work_/R_free_	0.164/0.203	0.259/0.283
*B*-factors (A^2^):		
Protein (all atoms)	24.0	58.1
GD1a/hSytI	29.0	73.1
Solvent	28.7	48.3
R.m.s.d. Bond lengths (Å)	0.001	0.002
R.m.s.d. Bond angles (°)	1.35	1.17
Ramachandran statistics:		
Favored (%)	96.26	95.32
Outliers (%)	0.23	0.25
**PDB ID**	6ZVM	6ZVN

^1^ Values in parentheses are for highest-resolution shell.

## Supplementary Materials

The following are available online at https://www.mdpi.com/2072-6651/12/9/603/s1: Complete sequences of constructs used in this study.
