# Supplementary material for: Structural and Biochemical Characterization of Botulinum Neurotoxin Subtype B2 Binding to Its Receptors
Source: Toxins (Basel). 2020 Sep 17;12(9):603. doi: 10.3390/toxins12090603 (PMC7551386; doi:10.3390/toxins12090603)
Supplement: Supplementary file 1 [file toxins-12-00603-s001.pdf]

Supplementary Materials: Structural and Biochemical Characterization of Botulinum Neurotoxin Subtype B2 Binding to Its Receptors

Jonathan R. Davies, Geoffrey Masuyer and Pål Stenmark

Complete sequences of protein constructs used in this study:

BONT binding domain (HC) used for all experiments

>His-FLAG-TEV-HC/B1

MGHHHHHHSGDYKDDDDKEDLYFQSHMNSEILNNIILNLRYKDNNLIDLSGYGAKVEVYDGVE
LNDKNQFKLTSSANSKIRVTQNQNIIFNSVFLDFSVSFWIRIPKYKNDGIQNYIHNEYTIINCMKNN
SGWKISIRGNRIIWTLIDINGKTKSVFFEYNIREDISEYINRWFFVTITNNLNNAKIYINGKLESNTDI
KDIREVIANGEIIFKLDGDIDRTQFIWMKYFSIFNTELSQSNIEERYKIQSYSEYLKDFWGNPLMYNK
EYYMFNAGNKNSYIKLKKDSPVGEILTRSKYNQNSKYINYRDLYIGEKFIIRRKSNSQSINDDIVRKE
DYIYLDFFNLNQEWRVYTYKYFKKEEEKLFLAPISDSDEFYNTIQIKEYDEQPTYSCQLLFKKDEEST
DEIGLIGIHRFYESGIVFEEYKDYFCISKWYLKEVKRKPYNLKLGCNWQFIPKDEGWTE

>His-FLAG-TEV-HC/B2

MGHHHHHHSGDYKDDDDKEDLYFQSHMNSEILNNIILNLRYRDNNLIDLSGYGANVEVYDGVE
LNDKNQFKLTSSTNSEIRVTQNQNIIFNSMFLDFSVSFWIRIPKYKNDGIQNYIHNEYTIINCIKNNS
GWKISIRGNRIIWTLTDINGKTKSVFFEYSIREDISDYINRWFFVTITNNSDNAKIYINGKLESNIDIK
DIGEVIANGEIIFKLDGDIDRTQFIWMKYFSIFNTELSQSNIKEIYKIQSYSEYLKDFWGNPLMYNKE
YYMFNAGNKNSYIKLKKDSSVGEILTRSKYNQNSNYINYRNLYIGEKFIIRRKSNSQSINDDIVRKE
DYIYLDFFNSNREWRVYAYKDFKEEEKKLFLANIYDSNEFYKTIQIKEYDEQPTYSCQLLFKKDEES
TDEIGLIGIHRFYESGIVLKDYKNYFCISKWYLKEVKRKPYNPNLGCNWQFIPKDEGWIE

Protein receptors used in the synaptotagmin-binding assays

>GST-hSytI

MSPILGYWKIKGLVQPTRLLLEYLEEKYEEHLYERDEGDKWRNKKFELGLEFPNLPYYIDGDVKLT
QSMAIIRYIADKHNMLGGCPKERAEISMLEGAVLDIRYGVSRIAYSKDFETLKVDFLSKLPEMLKM
FEDRLCHKTYLNGDHVTHPDFMLYDALDVVLYMDPMCLDAFPKLVCFKKRIEAIPQIDKYLKSSK
YIAWPLQGWQATFGGGDHPPKSDLIEGRGIPGHHHHHHHHGMVSESHHEALAAPPVTTVATVL
PSNATEPASPGEGKEDAFSKLKEKFMNELHK

>GST-hSytII

MSPILGYWKIKGLVQPTRLLLEYLEEKYEEHLYERDEGDKWRNKKFELGLEFPNLPYYIDGDVKLT
QSMAIIRYIADKHNMLGGCPKERAEISMLEGAVLDIRYGVSRIAYSKDFETLKVDFLSKLPEMLKM
FEDRLCHKTYLNGDHVTHPDFMLYDALDVVLYMDPMCLDAFPKLVCFKKRIEAIPQIDKYLKSSK
YIAWPLQGWQATFGGGDHPPKSDLIEGRGIPGHHHHHHHHGMRNIFKRNQEPIVAPATTTATM
PIGPVDNSTESGGAGESQEDMFAKLKEKLFNEINK

>GST-rSytII

MSPILGYWKIKGLVQPTRLLLEYLEEKYEEHLYERDEGDKWRNKKFELGLEFPNLPYYIDGDVKLT
QSMAIIRYIADKHNMLGGCPKERAEISMLEGAVLDIRYGVSRIAYSKDFETLKVDFLSKLPEMLKM
FEDRLCHKTYLNGDHVTHPDFMLYDALDVVLYMDPMCLDAFPKLVCFKKRIEAIPQIDKYLKSSK

YIAWPLQGWQATFGGGDHPPKSDLIEGRGIPGHHHHHHHHGMRNIFKRNQEPIVAPATTTATM
PLAPAAPADNSTESTGTGESQEDMFAKLKDKFFNEINK
